# Zika virus infection in pregnancy: a systematic review of disease course and complications

**DOI:** 10.1186/s12978-017-0285-6

**Published:** 2017-02-28

**Authors:** Ezinne C. Chibueze, Veronika Tirado, Katharina da Silva Lopes, Olukunmi O. Balogun, Yo Takemoto, Toshiyuki Swa, Amarjargal Dagvadorj, Chie Nagata, Naho Morisaki, Clara Menendez, Erika Ota, Rintaro Mori, Olufemi T. Oladapo

**Affiliations:** 1 Department of Health Policy, National Center for Child Health and Development, 2-10-1 Okura, Setagaya ku, 157-8535 Tokyo, Japan; 2 Department of Public Health Sciences, Global Health (IHCAR), Karolinska Institutet, Stockholm, Sweden; 3 Graduate School of Human Sciences, Osaka University, Osaka, Japan; 4 Department of Health Informatics, Kyoto University, Yoshida Konoe-cho, Syako-ku, Kyoto, Japan; 5 Department of Education for Clinical Research, National Center for Child Health and Development, Tokyo, Japan; 6 Department of Social Medicine, National Center for Child Health and Development, Tokyo, Japan; 7 Barcelona Institute for Global Health (ISGlobal)-Hospital Clinic-Universitat de Barcelona, Barcelona, Spain; 8 Department of Reproductive Health and Research World Health Organization, UNDP/UNFPA/UNICEF/WHO/World Bank Special Programme of Research, Development and Research Training in Human Reproduction (HRP), Geneva, Switzerland; 9 St. Luke’s International University, Graduate School of Nursing, Global Health Nursing, Tokyo, Japan

## Abstract

**Objectives:**

To characterize maternal Zika virus (ZIKV) infection and complement the evidence base for the WHO interim guidance on pregnancy management in the context of ZIKV infection.

**Methods:**

We searched the relevant database from inception until March 2016. Two review authors independently screened and assessed full texts of eligible reports and extracted data from relevant studies. The quality of studies was assessed using the Newcastle-Ottawa Scale (NOS) and the National Institute of Health (NIH) tool for observational studies and case series/reports, respectively.

**Results:**

Among 142 eligible full-text articles, 18 met the inclusion criteria (13 case series/reports and five cohort studies). Common symptoms among pregnant women with suspected/confirmed ZIKV infection were fever, rash, and arthralgia. One case of Guillain-Barré syndrome was reported among ZIKV-infected mothers, no other case of severe maternal morbidity or mortality reported. Complications reported in association with maternal ZIKV infection included a broad range of fetal and newborn neurological and ocular abnormalities; fetal growth restriction, stillbirth, and perinatal death. Microcephaly was the primary neurological complication reported in eight studies, with an incidence of about 1% among newborns of ZIKV infected women in one study.

**Conclusion:**

Given the extensive and variable fetal and newborn presentations/complications associated with prenatal ZIKV infection, and the dearth of information provided, knowledge gaps are evident. Further research and comprehensive reporting may provide a better understanding of ZIKV infection in pregnancy and attendant maternal/fetal complications. This knowledge could inform the creation of effective and evidence-based strategies, guidelines and recommendations aimed at the management of maternal ZIKV infection. Adherence to current best practice guidelines for prenatal care among health providers is encouraged, in the context of maternal ZIKV infection.

**Electronic supplementary material:**

The online version of this article (doi:10.1186/s12978-017-0285-6) contains supplementary material, which is available to authorized users.

## Plain english summary

*Aedes* mosquitoes transmit Zika virus (ZIKV) infection, its clinical presentation in humans is often mild or asymptomatic. Due to a marked increase in the number of symptomatic or suspected cases across continents, ZIKV infection was declared a Public Health Emergency of International Concern (PHEIC) on February 1, 2016. Pregnant women are at an exceptional risk of being affected with potential adverse effects. To describe maternal Zika virus (ZIKV) infection and complement the evidence base for the WHO interim guidance on pregnancy management and in the context of ZIKV infection, we did a systematic review.

We conducted database searches, independent screening of records and assessment of resulting full texts from which we extracted data. We further assessed the quality of the included studies.

Of the 142 eligible full-text articles, we extracted data from the 18 studies which met the inclusion criteria (13 case series/reports and five cohort studies). Among the pregnant women with suspected/confirmed ZIKV infection in these studies, fever, rash and joint-pain were the commonly reported symptoms. Neurological affectation (Guillain-Barre syndrome) was reported in one mother, no other cases of severe maternal illness or deaths were reported. Complications reported in association with maternal ZIKV infection included a broad range of fetal and newborn neurological and ocular abnormalities; fetal growth restriction, stillbirth, and perinatal death. Microcephaly was the primary neurological complication reported in eight studies, with an incidence of about 1% in one study.

In conclusion, knowledge gaps on the features characterising maternal ZIKV infection and its effects are evident. Further research and better reporting practices may help create effective strategies aimed at the management of maternal ZIKV infection and subsequent fetal complications. Pregnant women and health providers are encouraged to adhere to current best practice guidelines, in the context of maternal ZIKV infection.

## Background

Since the late 1940s, there have been reports of Zika virus (ZIKV) isolated from rhesus monkeys in Uganda, and human cases in Tanzania and Nigeria [1, 2]. At present, ZIKV has a broad distribution across sub-Saharan Africa, South-East Asia, and the Americas [3]. ZIKV belongs to the Flaviviridae family of viruses and is transmitted by *Aedes* mosquitoes.

In humans, ZIKV infection is often mild or asymptomatic. However, an exponential increase in the number of symptomatic or suspected cases across continents has intensified international concern [4]. Prior to 2007, few cases of ZIKV infection were reported [5]. Thereafter, an infectious outbreak affecting 74.6% of the Micronesian Yap population [6] and 32,000 people in the French Polynesian region in 2013–2014 were reported [7, 8]. In the latest outbreak in 2015, up to 1.3 million suspected cases were identified in Brazil over a 9-month period [9, 10]. In addition to transmission by *Aedes* mosquitoes, sexual transmission has also been reported [11, 12].

Despite the international efforts to curb its spread, ZIKV infection has expanded to 62 countries and territories, and is projected to increase due to potential climate change affecting the spread of the ZIKV *Aedes* vector [7, 13–15]. Notably in 2015, a 20-fold increase in the incidence of microcephaly relative to previous years was reported with the onset of ZIKV transmission in northeastern Brazil. This trend led to the World Health Organization (WHO) declaring a Public Health Emergency of International Concern (PHEIC) on February 1, 2016 [13, 16, 17].

Pregnant women are at exceptional risk of being infected with ZIKV infection and exhibiting potential adverse effects, including a wide range of congenital abnormalities associated with ZIKV infection *in utero*. Delayed or arrested neurological development and complications common in fetuses and neonates born to ZIKV-infected women, may drain family resources due to the need for special care. Perinatal deaths may further impose an emotional burden on affected families. The health systems in most ZIKV-affected areas have limited resources to manage the current outbreak and its consequences.

Many gaps in the knowledge regarding ZIKV infection exist, these include, the clinical spectrum of disease presentation in infected pregnant women, mechanisms of vertical transmission, and the risk of complications in mother, fetus and newborn. Also, the impact of ZIKV co-infection with other flaviviruses on pregnancy as well as the long-term consequence of ZIKV infection among infants and adults of reproductive age is unclear. As part of the process to complement the evidence base for WHO interim guidance on pregnancy care in the context of ZIKV infection and close the knowledge gaps, we conducted a systematic review.

## Methods

### Search strategy

We searched the following electronic databases: MEDLINE, EMBASE, CINAHL, World Health Organization Global Health Library (WHOGL), and Cochrane Central Register of Controlled Trials (CENTRAL) on March 3, 2016. An information specialist configured search terms for each database according to the level of term indexation. Search terms included flavivirus, chikungunya virus, fever, arbovirus, Zika, congenital malformations, cephalometry, and nervous system abnormalities as shown in Additional file 1). There were no dates, language or study design restrictions. We registered this review in PROSPERO, the international prospective register of systematic reviews of the University of York and the National Institute for Health Research, under the number CRD4201603969.

### Selection of studies

We considered all study designs including randomized controlled trials, prospective or retrospective cohorts, cross-sectional, case-control studies and case series/reports. Original reports, briefs, letters, editorials, correspondence and news reports were considered for inclusion. A minimum of two review authors assessed the title and abstract of references for relevance to review objectives. We evaluated the full text of potentially eligible records for inclusion relative to our review objectives. The review objectives included primary data on disease characteristics in ZIKV-infected pregnant women and their babies and the risk of adverse pregnancy outcomes. References of included reports were also searched for potentially eligible studies. Disagreements on the eligibility of reports, where present, were resolved by consensus with a third review author.

### Types of studies

We included all studies reporting on ZIKV infection and complications in pregnant women residing in or with a history of travel to areas of ongoing ZIKV transmission or recent ZIKV outbreaks. Studies comparing pregnant and non-pregnant women with ZIKV infection were considered for inclusion where available. We excluded any study or report on non-pregnant populations or lacking primary data.

### Types of participants

Pregnant women suspected of being at risk of, or diagnosed with ZIKV infection regardless of their location, or who have had sexual contact with a partner who (i) recently returned from an area of active ZIKV transmission, or (ii) was diagnosed with ZIKV infection.

### Data extraction and synthesis

Two review authors independently extracted and presented data based on the review objectives in an agreed data collection form. We summarized the data in a descriptive form (Table 1).

**Table 1:** Overview of publications by component of maternal, fetal, and newborn outcomes

Characteristics	Publications	Study designs	Countries	Reported findings	Other information	Knowledge gaps
Clinical manifestation: maternal, fetal and newborn	McCarthy M., [22] Ventura C.V., et al., [34] Ventura C.V., et al., [38] Villamil-Gómez W.E., et al. [24] Thomas D.L., et al. [25] Shuler-Faccini L., et al., [26] Reyna-Villasmil E., et al. [23] Oliveira Melo A., el atl., [30] Mlakar J., et al., [36] Meaney-Delman D., et al., [37] Kleber de Oliveira W., et al., [29] Calvet G., et al. [32] Brasil Martines R., et al., [27] Brasil P., et al., [33] Besnard M, et al., [31] de Paula Freitas B., et al. [28]	Retrospective cohort Case series Case series Case report Case report Retrospective cohort Case report Case report Case report Case series Retrospective + Prospect cohort Case series Case series Prospective cohort Case series Case series	Brazil Brazil Brazil Colombia Puerto Rico Brazil Venezuela Brazil Brazil USA Brazil Brazil Brazil Brazil Brazil Brazil	Cutaneous rash, maculopapular rash, fever, arthralgia, itch, myalgia, nausea or vomiting, bleeding, respiratory findings, conjunctivitis, malaise, headache, abdominal pain, chills, retroocular pain, edema in lower limbs, hemiparesis, asthenia, jaundice, lumbar pain	Besnard M, et al., reported mild pruritic rash with mild fever (37.5–38 °C) and without fever	- Selection bias based on symptoms suspicious of infection and observed in several studies limits our understanding of ZIKV infection in pregnancy. - Lack of a detailed history of infection to childbirth or related factors confines our of maternal ZIKV infection.
Clinical manifestations: not reported or vaguely reported	Cauchemez S., et al., [31] Oliveira Melo A., et al., [30]	Retrospective cohort Case report	French Polynesia Brazil	No rash, fever or other infection. Stated as “suffered from symptoms related to Zika virus infection.”		- Generalizations or vague reporting limited our understanding of associated ZIKV symptoms
Clinical manifestation: asymptomatic	Sarno M., et al., [35]	Case report	Brazil	No symptoms shown, the first indication of abnormal pregnancy was at ultrasound; intrauterine growth retardation at18 weeks’ gestation		The influence of an asymptomatic presentation on management modalities
Trimester infection	Ventura C.V., et al., [34] Ventura C.V., et al., [38] Sarno M., et al., [35] Villamil-Gómez W.E., et al., [24] Thomas D.L., et al., [25] Schuler-Faccini L., et al., [26] Reyna-Villasmil E., et al., [23] Mlakar J., et al., [36] Meaney-Delman D., et al., [37] Calvet G., et al., [32] Brasil Martines R., et al., [27] Besnard M., et al., [21] de Paula Freitas B., et al., [28]	Case series Case series Case report Case report Case report Retrospective cohort Case report Case report Case series Case series Case series Case series Case series	Brazil Brazil Brazil Colombia Puerto Rico Brazil Venezuela Brazil USA Brazil Brazil Brazil Brazil	50 First trimester 22 s trimester 25 Third Trimester 1 Post-partum	Cauchemez S., et al., [30] did not report trimester infection	- Timing of ZIKV infection is critical to maternal and fetal management, however, most studies made only generalised trimester-specific reports - This restricted our assessment of potential differences in disease susceptibility and progression during pregnancy
Effects on pregnancy complications (maternal)	Reyna-Villasmil E., et al., [23] Meaney-Delman D., et al., [37] Brasil Martines R., et al., [27] Brasil P., et al., [33]	Case report Case series Case series Prospective cohort	Venezuela USA Brazil Brazil	Guillain-Barré Syndrome; decreased muscle movements and difficulty speaking/swallowing, myalgia, fever, rash, and conjunctivitis for 10 days. Neurological examination showed logical alteration of cranial nerves and speech, decreased muscle strength and respiratory failure. Four miscarriages (first trimester) Two stillbirths (fetal deaths after 30 weeks of gestation)		- The isolated case of Guillain-Barré Syndrome and other neurological manifestations proposes a need for detailed neurological investigations in the context of ZIKV infection which was lacking - Proximal causes of stillbirths were not reported
Effects on pregnancy complications (fetus/newborn)	All publications except: Reyna-Villasmil E., et al., [23] Thomas D. L. et al., [25]	Case report Case report	Venezuela Puerto Rico	Microcephaly, hydraencephaly, macular alterations, optic abnormalities, intra-ocular calcification, cataracts, cerebral (intracranial) calcification, ascites and subcutaneous edema, coarse calcification, cerebellar involvement, severe arthrogryposis, severe central nervous system, affection and gross intrauterine growth retardation, ventriculomegaly	Brasil Martines, R., et al., [26] reported two fetal deaths Brasil, P., et al., 2016 reported two fetal deaths All fetal deaths occurred at >30 weeks gestation.	Causes of fetal deaths were unclear
Fetus alterations: frequencies/rates and absolute risk	Kleber de Oliveira W., et al., [29] Cauchemez S., et al., [31] Ventura C.V., et al., [34]	Retrospective + Prospective. cohort Retrospective cohort Case report	Brazil French Polynesia Brazil	Microcephaly had the highest prevalence in the Brazilian states of Pernambuco. Risk of microcephaly (estimated 1%) 95 cases (34–191) per 1000 women (first trimester) corresponding to a risk ratio of 53.4 (95% CI 6.5–1061–2) Severe ocular abnormalities when the infection occurs in the first or second trimester of pregnancy	^#^NR	Lack of clear estimates on the risk of fetal alterations in ZIKV infected pregnant women were observed
Postpartum clinical presentations (maternal)	Besnard M., et al., [21]	Case report	French Polynesia	Post-delivery mild pruritic rash, mild fever (37.5 – 38 °C) and myalgia	^#^NR	Limited information on maternal progression after childbirth for ZIKV-infected pregnant women
Postpartum clinical presentations (childbirth)	Brasil Martines, R., et al., [27] Besnard M., et al., [21]	Case report Case report	Brazil French Polynesia	Two newborns at 36 and 38 weeks’ gestation with microcephaly who died within 20 h of birth; 1 displayed a maculopapular rash on the 4th day after delivery and thrombocytopenia had severe hypotrophy.	^a^NR	Limited information on presentations at birth
Other tests	McCarthy M., [21] Ventura C.V., et al., [22] Ventura C.V., et al., [38] Sarno M., et al., [35] Villamil-Gómez W.E., et al., [24] Reyna-Villasmil E., et al., [23] Mlakar J., et al., [36] Calvet G., et al., [32] Brasil Martines R., et al., [27] Brazil P., et al., 2016 [33] de Paulas Freitas B., et al., 2016 [28]	Retrospective cohort Case series Case series Case report Case report Case report Case report Case series Case series Prospective cohort Case series	Brazil Brazil Brazil Brazil Colombia Venezuela Brazil Brazil Brazil Brazil Brazil	Negative serology for toxoplasmosis, rubella, cytomegalovirus, herpes simplex virus, or HIV, HTLV, HSV 1 &2, Rheumatoid fever, HBV, VDRL, EBV. Tests for dengue virus, chikungunya virus, toxoplasmosis, rubella virus, cytomegalovirus, Treponema pallidum and parvovirus B19, syphilis.	Ventura, C.V., et al., [33] had positive IgG and negative IgM ELISA results for toxoplasmosis, rubella virus and cytomegalovirus Villamil-Gómez, W.E., et al., [23] reported one positive (isolating Escherichia coli) trichomonas trophozoites and three positives for Toxoplasma IgG and one for rubella. Brazil, P., et al., 2016 reported immunity to rubella and cytomegalovirus Besnard, M., et al., [20] reported dengue negative test results.	- For studies which reported on the presence of coinfections, potential synergy due to the presence of immunity and/or seropositivity to other viruses could not be ascertained - The effect of coinfections on disease course, consequent maternal, fetal and neonatal outcomes is unknown

*ZIKV* Zika virus, ^a^ *NR* Not Reported, *HTLV* Human T-lymphotropic virus, *HSV* Herpes simplex virus, *HBV* Hepatitis B virus, *VDRL* Venereal disease research laboratory test, *EBV* Epstein-Barr Virus, *IgG* Immunoglobulin G, *IgM* Immunoglobulin M, Case report – case description (i.e. symptoms, cause or outcomes, etc.) of an individual patient, Case series – case descriptions (i.e. symptoms, cause or outcomes, etc.) of several patients

We extracted information on study design, sample size, history of maternal and fetal ZIKV infection, symptoms, presence and type of clinical features, pregnancy-specific complications during pregnancy, childbirth or postpartum period. The absolute risk of microcephaly and other birth defects was also recorded where available.

### Risk of bias assessment

Two review authors conducted independent assessments on the quality of included studies. Where discordance in ranking was observed, this was resolved by consensus or discussion with a third review author. For individual case series/reports, a quality assessment of the National Institute of Health Tool (NIH) [18] was applied. This tool includes questions based on nine criteria to which either of the binary answers (Yes/No) was allotted. Based on the number of ‘Yes’ answers, a rating of good (7 – 9), fair (4 – 6) or poor (≤3) was allocated to the individual study and differences in quality ratings amended by consensus. Studies for which the criteria were irrelevant were labelled ‘not applicable’ and ‘cannot determine’ if such information was lacking in the study.

For observational studies, we applied the Newcastle-Ottawa Scale [18] which consisted of lead questions aimed at assessing three domains – selection, comparability, and outcome. For each outcome, a maximum of three points could be allotted as previously described [20].

## Results

### Search results

**Fig. 1:**
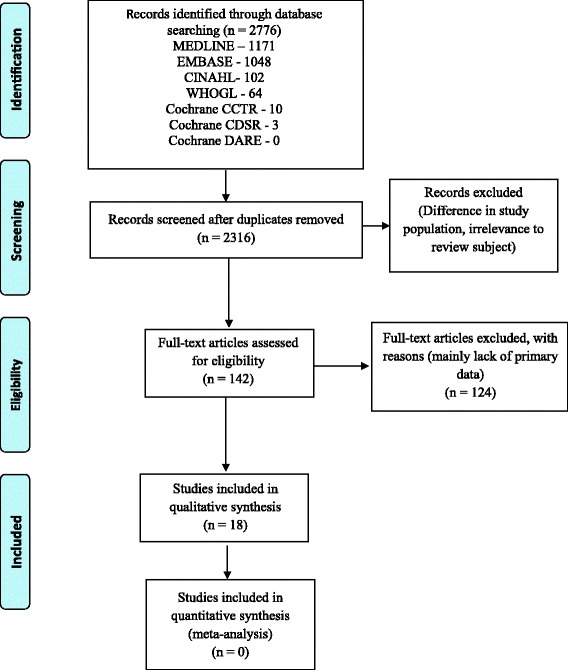
Flow diagram of search results and study selection

Search strategies identified 2316 records as shown in the PRISMA flow chart in Fig. 1. Based on the title and abstract screening, 2174 records were excluded due to lack of primary data and/or irrelevance to review objective. Of the resulting 142 eligible full texts, 18 studies met our inclusion criteria (reasons for exclusion are outlined in Additional file 2).

### Characteristics of included studies

All but one study were published in 2016 [21]. The studies were conducted largely in South America: Brazil [11], Colombia [1], Puerto Rico [1], and Venezuela [1]. Other studies were conducted in France [1], Slovenia [1] and the USA [2]. In all studies, pregnant women were either living in areas of ongoing transmission [21–35], had resided in [36], or travelled [37] to ZIKV-affected areas during pregnancy.

Regarding study design, there were 13 case series/reports [21, 23–27, 28, 30, 32, 34–35] and five observational studies [22, 26, 29, 31, 33]. The observational studies were either prospective [33], retrospective [22] or mixed [29, 31] in design. All presented information on clinical manifestations, pregnancy-specific complications and diagnoses in women and their fetuses/newborns.

Diagnostic tests to confirm the presence of ZIKV infection in pregnant women included reverse transcription polymerase chain reaction (RT-PCR) for ZIKV nucleic material [21, 25, 29, 30, 33, 35, 37] and tests on serum [21, 24], breastmilk [21], amniotic fluid [37] and urine [24] samples. IgG and IgM antibody tests for viral ZIKV exposure were also conducted [24, 28, 33, 35]. Eleven of the 18 included studies conducted serological tests to exclude other infections such as dengue [21, 27, 32, 33], chikungunya [32], toxoplasmosis [22, 24, 27, 28, 32, 34], rubella [22–24, 27, 28, 32, 33], cytomegalovirus [22–24, 27, 28, 32, 33], herpes simplex virus [22, 24, 27, 28, 32], HIV [22, 24, 27, 28, 32, 35], Human T-cell lymphotrophic virus [35], Hepatitis C Virus [35] and Hepatitis B Virus [24].

For fetal imaging, ultrasound [24, 30, 32, 35], computed tomography scanning for brain calcifications [25] and magnetic resonance imaging [37] were employed.

In some studies, ocular examinations were also conducted for the mother [34] and newborn [22, 34].

### Characteristics of maternal ZIKV infection

#### Symptoms/signs/complications

No study suggested a higher risk of acquiring ZIKV infection in pregnant women compared to the non-pregnant population, after exposure to *Aedes* mosquitoes. Most studies only presented fetal and newborn features without further information on how these varied with the gestational age at the time of maternal ZIKV infection.

Rash was the most common sign of ZIKV infection in pregnant women in 15 of 18 included reports [21–29, 32–34]. Two of the remaining three studies (one observational study, one case series) did not report clinical manifestations during pregnancy as they focused on the newborns of ZIKV-infected mothers [30, 31]. Rash was absent in the third study as the mothers were asymptomatic [35]. In seven reports, the rash was further qualified as ‘a pruritic cutaneous rash with itching in the back and hands’, ‘generalised, descending macular’ or ‘generalised maculopapular’ [21, 28, 33], ‘petechial’ or ‘generalised’ [21, 28, 32, 33, 36]. Only one case-series reported on symptom occurrence after delivery in two ZIKV-infected pregnant women [21]. A 4-day duration of persistent rash in one pregnant woman and a pruritic rash on the third day after childbirth in the second case was observed. In the former, the rash started 2 days before and ended 2 days after delivery of a healthy infant. In the latter case, a mild fever and myalgia were also reported.

Other reported signs and symptoms included fever [22–24, 27–29, 32, 33], chills [23, 24], malaise [24, 29, 34], arthralgia [22, 24, 28, 29, 33, 34], myalgia [23, 24, 29, 32, 33], myotonia [23], asthenia [24], jaundice [24], paraesthesia [33], hemiparesis [24], headache [22, 28], conjunctivitis or conjunctival injections [23, 33], lymphadenopathy [33], pain (eye, abdominal, lumbar, pelvis, body and joint) [24, 29], anaemia [24], edema in lower limbs [24], nausea [25, 33], vomiting [33], dermal bleeding [33] and ‘respiratory findings’ [33].

The grade of fever was provided in three reports, a low grade fever of 37.5 to 38.0 °C in two reports [21, 33] and a “high fever” [36] in the third. Fever was absent in ZIKV-infected pregnant women in two case reports [35].

An observational study reported a significantly higher frequency of lymphadenopathy and conjunctival injections in ZIKV-infected compared with uninfected pregnant women [33].

Twelve studies provided information on trimester-specific occurrence of symptoms [21, 23, 24, 26–29, 32, 34]. Symptoms were observed more commonly in the first trimester [24, 26–28, 32, 34]. Among the ZIKV-infected pregnant women in individual reports, seven studies reported no signs or symptoms [22, 24, 26, 28, 34, 35].

Neurological complications were reported in ZIKV-infected pregnant women in one case report [23] and one observational study [33]. Guillain-Barré syndrome (GBS) at 28 weeks gestation in addition to other typical ZIKV signs and symptoms were reported in one case report only [23]. The patient had respiratory distress that prompted intensive care unit (ICU) admission. She fully recovered within 3 weeks and gave birth to a normal infant at 39 weeks of gestation.

Preterm birth necessitating emergency caesarean delivery was reported in two observational studies [27, 33] and one case series [21]. In the two observational studies, intrauterine growth restriction (IUGR) was an underlying complication. In one of the studies [33], the two preterm births reported were due to IUGR accompanied by oligohydramnios, macular hypoplasia, and placental insufficiency in one fetus and anhydramnios in the other.

Miscarriages were reported in five ZIKV-infected pregnant women in two case series [27] and one observational study [33]. Stillbirths were also recorded in three pregnant women in one observational study and one case report [33, 35].

### Characteristics of fetuses/newborns of ZIKV infected pregnant women

#### Fetuses

##### Complications

A wide spectrum of complications was reported in association with maternal ZIKV infection (Table 2). Microcephaly visible on fetal ultrasound was the main neurologic complication in two observational studies and six case series/reports [24, 27, 29, 30, 32, 33, 35]. Among them, ocular abnormalities [22, 28, 30, 32, 34], cerebral calcifications and cerebellar abnormalities were commonly reported [24, 30, 32–35]. In one observational study of 35 infants with microcephaly [26], 11 fetuses had intra-uterine brain injury accompanied by stunting of cerebral growth prior to birth. Cerebral abnormalities included brain atrophy, absent corpus callosum, and ventriculomegaly.

**Table 2:** Methodological quality assessment for case reports using the National Institute

Study ID	Ventura C. V [34, 38]	Ventura C. V [34, 38]	Sarno M [35]	Villamil Gomez W. E [24]	Thomas D. L [25]	Reyna-Villasmil [23]	Oliveira Melo [30]	Mlakar [36]	Meaney-Delman [37]	Calvet G [32]	Brasil Martines [27]	Besnard M [21]	de Paula Freitas [28]
1. Was the study question or objective clearly stated?	YES	YES	YES	YES	YES	YES	NO	YES	YES	YES	YES	YES	YES
2. Was the study population clearly and fully described, including a case definition?	NO	YES	YES	YES	NO	YES	NO	YES	YES	YES	NO	YES	YES
3. Were the cases consecutive?	YES	YES	NA	YES	NO	NA	YES	NA	YES	YES	YES	YES	YES
4. Were the subjects comparable?	YES	YES	NA	YES	NO	NA	YES	NA	YES	YES	YES	YES	YES
5. Was the exposure clearly described?	YES	YES	YES	YES	YES	YES	YES	YES	YES	YES	NO	YES	YES
6. Were the outcome measures clearly defined, valid, reliable, and implemented consistently across all study participants?	YES	YES	YES	YES	YES	YES	YES	YES	YES	YES	YES	YES	NO
7. Was the length of follow-up adequate?	NO	YES	YES	YES	^a^CD	YES	^a^CD	YES	^a^CD	YES	NO	NO	NA
8. Were the statistical methods well-described?	NO	YES	NO	NO	NO	NO	^b^NA	NO	^b^NA	^b^NA	^b^NA	^b^NA	YES
9. Were the results well-described?	YES	YES	YES	YES	NO	YES	YES	YES	NO	YES	YES	YES	YES
Quality Rating (Good, Fair, or Poor)	FAIR	GOOD	GOOD	GOOD Letter to editor	POOR Selective reporting	GOOD Letter to editor	FAIR	GOOD	FAIR	GOOD	FAIR	GOOD	GOOD

^a^ *CD* cannot determine ^b^ *NA* Not available

Ocular abnormalities included intraocular calcifications and microphthalmia [30, 32].

In fetuses of ZIKV-infected pregnant women, placental and umbilical [32, 33] and amniotic fluid [33] abnormalities were reported.

IUGR was reported in five studies (two observational, three case-series/reports) [21, 33] and stillbirths in two studies [33, 35]. Despite normal fetal biometrics at 14 weeks in one case of an asymptomatic ZIKV-infected pregnant woman, IUGR was detected at 18 weeks and accompanied by other neurological abnormalities including microcephaly, hydranencephaly, hydrops fetalis and eventual stillbirth [35].

Fetal deaths were reported in two studies (one case series and one observational study), these deaths occurred in four fetuses at > 30 weeks gestation [27, 33]. Elective terminations of pregnancies were conducted for seven pregnant women. However, the gestational age or trimester of termination was unknown [31].

#### Newborns

##### Symptoms/signs

Rash described as ‘transient isolated diffuse’ was reported in an infant on the fourth day post-delivery [21]. Other symptoms reported included conjunctivitis or conjunctival injections [24, 28].

##### Complications

Similar to fetuses, microcephaly was observed at birth in 11 studies (four observreports)/reports) [22, 26–30, 32–34] with accompanying ocular and brain abnormalities. In four studies, microcephaly that was observed at birth was preceded by a fetal diagnosis of IUGR [25, 32, 34, 35]. Ocular abnormalities included focal pigment mottling, chorioretinal macular atrophy, optic nerve abnormalities, cataracts, intra-ocular calcifications, microphthalmia, conjunctival injections, optic disc cupping, lens subluxation in addition to bilateral iris coloboma, foveal reflex loss, macular hypoplasia and scarring [22, 28, 34, 38].

Musculoskeletal abnormalities reported included clubfoot [33] and severe arthrogryposis [30, 32] in two case series. In one case report, ‘arthrogryposis’ was also reported at post-mortem examination [35].

Four studies reported the birth of healthy infants [23, 33, 37] and one study reported early neonatal deaths in three infants with microcephaly within 20 h of delivery [27].

#### Absolute risk of fetal microcephaly (and other birth defects) in women with ZIKV infection

No report provided detailed information from which factors related to ZIKV infection could be described. One observational study provided a trimester-specific modelling estimate risk for microcephaly in first [95 cases (95% confidence interval (CI) 34-191)], second [84 cases (95% CI 12–196)] and third [0 case (95% CI 0–251)] trimesters, per 10,000 ZIKV-infected pregnant women [30]. The baseline prevalence for the risk of microcephaly were 2% (0 – 8), corresponding to a risk ratio of 53.4 (95% CI 6.5 – 1061.2) in the first trimester; 4% (95% CI 0 – 12), corresponding to a risk ratio of 23.2 (95% CI 1.4 – 407.8) in the second trimester; and 10% (95% CI 3 – 18), corresponding to a risk ratio of 0 (95% CI 0 – 49.3) in the third trimester.

Fetal abnormalities, including microcephaly, were detected in women who underwent an ultrasound at time-points ranging between 26 and 30 weeks [30, 35, 36].

#### Impact of ZIKV co-infection in pregnancy

There was a lack of information on co-infection with other flaviviruses and common congenital infections in some studies. Serological tests were conducted in studies to exclude possible co-infections and/or antibody tests for previous exposure to other related flaviviruses.

Eleven reports assessed for the presence of co-infections, toxoplasmosis [22, 24, 27, 28, 32–35, 38], dengue [21, 27, 32], chikungunya [27, 32], HIV [22, 24, 27, 28, 32, 33, 35, 38], hepatitis B virus (HBV) [24], hepatitis C virus (HCV) [35], cytomegalovirus (CMV) [22, 24, 27, 28, 32, 33, 35, 38], herpes simplex virus (HSV) [22, 24, 27, 28, 32, 38], Epstein-Barr virus (EBV) [24], rubella [22, 24, 27, 28, 32, 33, 35, 38], human T lymphotrophic virus (HTLV) [35], parvovirus B19 [32], syphilis [24, 28, 32] and rheumatoid fever [24].

Four reports assessed previous exposure to other infections based on the presence of IgG [24, 28, 33, 35] and IgM [28, 35] antibody positivity to cytomegalovirus, rubella and toxoplasmosis [24, 33, 35] infections. IgG positivity to infections indicating exposure to dengue virus in 88% of 88 pregnant women was reported in one observational study [33], and toxoplasmosis and rubella in three of 28 women [24] in two case series/report.

### Risk of bias assessment

We judged the overall risk of bias as fair or good for all the included case series/reports except one, which we rated as poor [25] due to selective reporting (Table 2).

Four of five observational studies included in the review were assigned an average quality rating of four to five stars based on the NOS. One observational study [22] was assigned a very low quality rating of one star as it provided sparse information (Table 3).

**Table 3:** Methodological quality assessment for cohort studies using the Newcastle-Ottawa Scale (NOS)

New Castle Ottawa Scale					
Study ID	McCarthy, M [22] (retrospective cohort study)	Schuler-Faccini [26] (retrospective cohort study)	Kleber de Oliveira [29] (retrospective cohort study)	Cauchemez S [31] (retrospective cohort study)	Brasil Patricia [33] 2016 (prospective cohort study)
Representativeness of exposed cohort	Not population based (Gotten from a teaching hospital in Salvador, one state)	^a^(infants born in eight of Brazil’s states 26 states and reported to the registry)	^a^(infants born in three of Brazil’s states 26 states and reported to the registry)	^a^(datasets from the French Polynesia Zika virus outbreak)	Not population based (Gotten from centers in Rio de Janeiro, one state)
Truly representative of the average woman^a^					
Somewhat representative of the average woman^a^					
Selected group of users					
No description of the derivation of the cohort					
Selection of non-exposed cohort	No non-exposed cohort	No non-exposed cohort	Only one cohort of exposed individuals	Only one cohort of exposed individuals	Only one cohort of exposed individuals
Drawn from the same community as the exposed cohort^a^					
Drawn from a different source					
No description of the derivation of the non-exposed cohort					
Ascertainment of exposure	No description	No description	^a^ (Registry)	^a^(Serological and surveillance data)	^a^(data gotten from clinical and US data)
Secure records (e.g., surgical records)^a^					
Structured interview^a^					
Written self-report					
No description					
Demonstration that outcome of interest not present at study start	^a^ (Yes, MCP or familial history was excluded)	No	No	No	^a^(Yes. No women had had diagnoses of fetal malformations in the current pregnancy before enrollment)
Yes^a^					
No					
Comparability of cohorts on the basis of the design or analysis	Only one cohort of exposed individuals	Only one cohort of exposed individuals	Only one cohort of exposed individuals	Only one cohort of exposed individuals	Only one cohort of exposed individuals
Study controls for gestational age and/or birth weight^a^					
Study controls for any additional factor^a^					
Assessment of outcome	No description	^a^(Record (registry) linkage implied)	^a^(Record linkage)	^a^(Record linkage from datasets)	^a^(Record linkage and self-report as they were followed up weekly by telephone)
Independent blind assessment^a^					
Record linkage^a^ Self-report					
No description					
Follow-up long enough for outcomes to occur	No	^a^(Yes, for mothers of infants with MCP^b^ born during August to October 2015)	^a^(Yes, from January 1, 2015–January 7, 2016)	^a^(Yes, over a 23-month study period)	^a^(Yes, women were followed up from September 2015 through February 2016)
Yes^a^					
No					
Adequacy of follow-up of cohorts	Not reported	^a^(all subjects accounted for)	Not reported	^a^(All subjects accounted for)	^a^(all subjects accounted for)
Complete follow-up – all subjects accounted for^a^					
Subjects lost to follow-up unlikely to introduce bias or description provided of those lost^a^					
No statement					
Total number of stars	1 star	4 stars	4 stars	5 stars	5 stars

^a^refers to a ‘star system’; ^b^MCP microcephaly

## Discussion

Our systematic review describes the complications to fetuses and newborns have been reported during ZIKV infection in pregnant women. However, no study clearly described the history of maternal ZIKV infection and its effects on newborns of affected mothers. A lack of information on factors related to ZIKV infection in pregnancy further limits our understanding. Most studies were conducted in 2016, these studies focused on the presenting symptoms and associated complications in pregnant women and their fetuses/newborns. Relative to non-pregnant women or the general population [12, 39], symptoms and complications of ZIKV infection were comparable.

Maternal ZIKV infection tended to have more adverse effects on the fetus and the infant than on the mother, as most maternal symptoms were self-limiting. A broad spectrum of clinical presentation was observed in fetuses and newborns of ZIKV-infected mothers, ranging from normal to abnormal ultrasound findings during pregnancy, such as healthy newborns or newborns with an abnormality. A similar pattern was observed in ZIKV-infected pregnant women, ranging from symptomatic and asymptomatic clinical presentation to uncomplicated deliveries in most studies. Severe pregnancy-specific complications were uncommon and no maternal deaths were recorded. This highlights a challenge for both health providers and mothers at risk because of the increased likelihood of missed opportunities due to the frequent asymptomatic presentation.

The diagnostic accuracy of tests employed in the ZIKV context is still unclear [40] because existing studies are based on epidemiological models. In some women, ZIKV positivity was confirmed later in pregnancy, although the standard RT-PCR tests done in early trimesters were negative. Fetal ultrasound tests that were negative in the first and second trimesters were positive in late pregnancy stage [35]. The absolute risk of developing fetal abnormalities is also unclear, but microcephaly is unlikely being a rare condition.

ZIKV shares the same vector with other arboviruses including chikungunya and dengue. TORCH (Toxoplasmosis, Other (syphilis, varicella-zoster, parvovirus B19), Rubella, Cytomegalovirus (CMV), and Herpes) infections have been associated with congenital malformations including central nervous system anomalies. In the included reports, the absence of co-infection with or previous exposure to other viruses was established in most of the pregnant women. In some studies, IgG-positivity to dengue and toxoplasma were observed. Immunity to rubella and CMV were reported in some women with poor outcomes. Potential synergy due to the presence of immunity and/or seropositivity to other viruses could not be ascertained as clinical presentation varied from the absence of symptoms to the presence of typical symptoms seen in ZIKV-infected pregnant women. In one study, about half of the pregnant women who presented with rash had lymphadenopathy [33], a condition commonly observed in dengue infection due to lymphatic infiltration [41]. Although inconclusive, the history of lymphadenopathy in some pregnant women in this study in addition to the presentation of rash may show a possible role for dengue in extending the spectrum of maternal or fetal presentation. Indirect evidence from the history of viral infections that share similar characteristics and complications with ZIKV, such as CMV, may be important in understanding ZIKV infection.

In some studies, sample selection was based on the presenting clinical features. Limitation of the study population to only pregnant women presenting with features suggestive of ZIKV infection such as rash and fetuses with microcephaly as seen in some reports could be misleading. The rigorous search strategy, the inclusion of foreign language studies and exclusion of overlapping studies add to the strength of this review.

The broad spectrum of clinical presentation and complications in fetuses and newborns of ZIKV-infected pregnant women provides an insight into the potential liability for affected women, their families and health systems in resource-constrained settings. The possibility of an asymptomatic presentation proposes an emphasis on routine antenatal care and attention to signs of fetal brain abnormalities by health providers in pregnant women with molecular or epidemiological links to ZIKV infection.

Ongoing research on live animal models may help to understand the pathogenesis of ZIKV, with a potential for application to exposed pregnant cohorts. The rapid rate of emerging data from research on Zika virus infections in pregnancy and recent years proposes a need for frequent updates. As newer studies become available, further research on adverse pregnancy outcomes and implications on long-term consequences of ZIKV during pregnancy will further enable early diagnosis and better management modalities in provider care. Furthermore, personal protective measures should be encouraged.

## Conclusions

This review highlights key evidence gaps that need to be urgently prioritized by the international community, more so, with the wide variations in fetal and newborn presentations/complications associated with prenatal ZIKV infection. Further research and comprehensive reporting of maternal ZIKV infection and fetal/neonatal complications may provide a better understanding of ZIKV infection in pregnancy and its attendant maternal/fetal complications. Such knowledge could inform the creation of effective and evidence-based strategies, guidelines, recommendations and health policies aimed at the management of maternal ZIKV infection.

Adherence to current best practices guidelines for prenatal care among health providers is encouraged. These include appropriate notification of suspected cases to the responsible authorities, long-term evaluation, monitoring and follow-up for newborns and infants exposed to ZIKV infection by a multidisciplinary team of health providers, more so, with ongoing variations in presentation and distribution of maternal ZIKV infection.

## Additional files

Additional file 1: Database search strategies for prenatal diagnosis of microcephaly in the context of ZIKV infection on March 3^rd^ 2016. (DOCX 29 kb)
Additional file 2: List of excluded studies ZIKV & pregnancy RHER1. (DOCX 31 kb)

## Abbreviations

CENTRAL: Cochrane Central Register of Controlled Trials
CINAHL: Cumulative index of nursing and allied health literature
EMBASE: Excerpta medica database
IUGR: Intrauterine growth restriction
MEDLINE: Medical literature analysis and retrieval system online
PRISMA: Preferred reporting items for systematic reviews and meta-analyses
TORCH: Toxoplasmosis, other (syphilis, varicella-zoster, parvovirus B19), rubella, cytomegalovirus (CMV), and herpes
WHOGL: World health organization global health library
ZIKV: Zika virus
