# Supplementary material for: Zika virus infection in pregnancy: a systematic review of disease course and complications
Source: Reprod Health. 2017 Feb 28;14:28. doi: 10.1186/s12978-017-0285-6 (PMC5330035; doi:10.1186/s12978-017-0285-6)
Supplement: Additional file 1: — Database search strategies for prenatal diagnosis of microcephaly in the context of ZIKV infection on March 3rd 2016. (DOCX 29 kb) [file 12978_2017_285_MOESM1_ESM.docx]

Additional file 1

| **Database search strategies for prenatal diagnosis of microcephaly in the context of ZIKV infection on March 3^rd^ 2016** | | |
| --- | --- | --- |
| **No** | **MEDLINE** | **Results** |
| 1 | exp Flavivirus/ | 16541 |
| 2 | exp Flavivirus Infections/ | 20393 |
| 3 | (zika or flavivi* or "flavi vi*" or dengue or (encephalitis adj3 (japan* or "st louis" or "tick borne")) or "west nile fever*" or "yellow fever*").mp. | 34755 |
| 4 | or/1-3 | 35485 |
| 5 | Chikungunya Virus/ | 1534 |
| 6 | Chikungunya Fever/ | 584 |
| 7 | chikungunya.mp. | 2689 |
| 8 | Arboviruses/ | 3169 |
| 9 | arbovirus*.mp. | 8293 |
| 10 | or/5-9 | 10400 |
| 11 | or/4,10 | 42307 |
| 12 | exp "Congenital, Hereditary, and Neonatal Diseases and Abnormalities"/ | 1E+06 |
| 13 | exp "Embryonic and Fetal Development"/ | 229585 |
| 14 | exp Embryonic Structures/ | 391915 |
| 15 | exp Nerve Growth Factors/ | 41137 |
| 16 | Cephalometry/ | 24107 |
| 17 | Maternal Exposure/ | 6172 |
| 18 | exp Pregnancy/ | 784084 |
| 19 | Pregnant Women/ |  |
| 20 | Prenatal Care/ | 21837 |
| 21 | exp Prenatal Diagnosis/ | 63551 |
| 22 | or/12-21 | 2E+06 |
| 23 | 11 and 22 | 1607 |
| 24 | (microcephal* or microlissencephal* or anencephal* or ((congenital or brain or cerebral or "white matter" or nerv* or neur*) adj3 (malformation* or abnormalit* or defect* or calcification or development* or growth))).mp. | 266351 |
| 25 | (cephalometr* or (head adj3 (circumference* or size))).mp. | 31460 |
| 26 | (prenatal or antenatal or fetus or fetal or foetus or foetal or gestation* or intrauter* or pregnan* or "expectant mother*").mp. | 1E+06 |
| 27 | embryo*.mp. | 376522 |
| 28 | stillbirth*.mp. | 10082 |
| 29 | neurotroph*.mp. | 34460 |
| 30 | or/24-29 | 2E+06 |
| 31 | 11 and 30 | 1811 |
| 32 | 31 not medline.st. | 124 |
| 33 | exp Nervous System Malformations/cn, em, ep, et [Congenital, Embryology, Epidemiology, Etiology] | 7727 |
| 34 | TORCH.mp. | 930 |
| 35 | Toxoplasma/ | 11353 |
| 36 | exp Toxoplasmosis/ | 17866 |
| 37 | exp Viruses/ | 673651 |
| 38 | exp Virus Diseases/ | 783448 |
| 39 | Aedes/ | 11697 |
| 40 | or/34-39 | 1E+06 |
| 41 | 33 and 40 | 243 |
| 42 | or/23,32,41 | 1959 |
| 43 | remove duplicates from 42 | 1954 |
| 44 | 43 not (animals.sh. not (humans.sh. or 39)) | 1216 |
| 45 | limit 44 to (comment or editorial or news or newspaper article) | 45 |
| 46 | 44 not 45 | 1171 |

| **No.** | **EMBASE** | **Results** |
| --- | --- | --- |
| #1 | 'flavivirus'/exp | 23321 |
| #2 | 'flavivirus infection'/exp | 22456 |
| #3 | zika OR flavivi* OR 'flavi vi*' OR dengue OR encephalitis NEAR/3 (japan* OR 'st louis' OR 'tick borne') OR 'west nile fever*' OR 'yellow fever*' | 40882 |
| #4 | 'chikungunya virus'/de | 2271 |
| #5 | 'chikungunya'/de | 1519 |
| #6 | chikungunya | 3625 |
| #7 | 'arbovirus'/de | 4180 |
| #8 | arbovirus* | 7688 |
| #9 | #1 OR #2 OR #3 OR #4 OR #5 OR #6 OR #7 OR #8 | 50455 |
| #10 | 'congenital malformation'/exp | 713508 |
| #11 | 'prenatal development'/exp | 201031 |
| #12 | 'embryo (anatomy)'/exp | 213844 |
| #13 | 'neurotrophic factor'/exp | 60485 |
| #14 | 'cephalometry'/de | 20598 |
| #15 | 'pregnancy'/exp | 649044 |
| #16 | 'expectant mother'/de | 288 |
| #17 | 'prenatal care'/exp | 120829 |
| #18 | 'prenatal diagnosis'/exp | 87727 |
| #19 | microcephal* OR microlissencephal* OR anencephal* OR (congenital OR brain OR cerebral OR 'white matter' OR nerv* OR neur*) NEAR/3 (malformation* OR abnormalit* OR defect* OR calcification OR development* OR growth) | 535932 |
| #20 | cephalometr* OR head NEAR/3 (circumference* OR size) | 33737 |
| #21 | prenatal OR antenatal OR fetus OR fetal OR foetus OR foetal OR gestation* OR intrauter* OR pregnan* OR 'expectant mother*' OR stillbirth* | 1212384 |
| #22 | embryo* | 515358 |
| #23 | neurotroph* | 53990 |
| #24 | #10 OR #11 OR #12 OR #13 OR #14 OR #15 OR #16 OR #17 OR #18 OR #19 OR #20 OR #21 OR #22 OR #23 | 2536003 |
| #25 | #9 AND #24 | 2579 |
| #26 | 'nervous system malformation'/exp/'congenital disorder','etiology','epidemiology' | 18065 |
| #27 | torch | 1438 |
| #28 | 'toxoplasma'/exp | 16001 |
| #29 | 'toxoplasmosis'/de | 19821 |
| #30 | 'virus'/exp | 893164 |
| #31 | 'virus infection'/exp | 964171 |
| #32 | 'aedes'/exp | 13176 |
| #33 | #27 OR #28 OR #29 OR #30 OR #31 OR #32 | 1492147 |
| #34 | #26 AND #33 | 513 |
| #35 | #25 OR #34 | 3081 |
| #36 | #35 AND [humans]/lim NOT ([animals]/lim NOT #32) | 1307 |

| **No** | **CENTRAL** | **Results** |
| --- | --- | --- |
| #1 | MeSH descriptor: [Flavivirus] explode all trees | 124 |
| #2 | MeSH descriptor: [Flavivirus Infections] explode all trees | 241 |
| #3 | zika or flavivi* or "flavi vi*" or dengue or (encephalitis near/3 (japan* or "st louis" or "tick borne")) or "west nile fever*" or "yellow fever*":ti,ab,kw | 536 |
| #4 | #1 or #2 or #3 | 536 |
| #5 | MeSH descriptor: [Chikungunya virus] this term only | 4 |
| #6 | MeSH descriptor: [Chikungunya Fever] this term only | 2 |
| #7 | chikungunya:ti,ab,kw | 14 |
| #8 | MeSH descriptor: [Arboviruses] this term only | 1 |
| #9 | arbovirus*:ti,ab,kw | 6 |
| #10 | #5 or #6 or #7 or #8 or #9 | 20 |
| #11 | #4 or #10 | 553 |
| #12 | MeSH descriptor: [Congenital, Hereditary, and Neonatal Diseases and Abnormalities] explode all trees | 15296 |
| #13 | MeSH descriptor: [Embryonic and Fetal Development] explode all trees | 3554 |
| #14 | MeSH descriptor: [Embryonic Structures] explode all trees | 2655 |
| #15 | MeSH descriptor: [Cephalometry] this term only | 592 |
| #16 | MeSH descriptor: [Maternal Exposure] this term only | 46 |
| #17 | MeSH descriptor: [Prenatal Care] this term only | 1197 |
| #18 | MeSH descriptor: [Prenatal Diagnosis] explode all trees | 1021 |
| #19 | (microcephal* or microlissencephal* or anencephal* or ((congenital or brain or cerebral or "white matter" or nerv* or neur*) near/3 (malformation* or abnormalit* or defect* or calcification or development* or growth))):ti,ab,kw | 3633 |
| #20 | (cephalometr* or (head near/3 (circumference* or size))):ti,ab,kw | 1385 |
| #21 | (prenatal or antenatal or fetus or fetal or foetus or foetal or gestation* or intrauter* or pregnan* or "expectant mother*" or stillbirth*):ti,ab,kw | 37768 |
| #22 | embryo*:ti,ab,kw | 3817 |
| #23 | neurotroph*:ti,ab,kw | 587 |
| #24 | #12 or #13 or #14 or #15 or #16 or #17 or #18 or #19 or #20 or #21 or #22 or #23 | 55078 |
| #25 | #11 and #24 | 13 |
| #26 | MeSH descriptor: [Nervous System Malformations] explode all trees and with qualifier(s): [Etiology - ET] | 9 |
| #27 | #25 or #26 | 22 |

|  | |  |
| --- | --- | --- |
| **Search ID#** | **CINAHL** | **Results** |
| S34 | S26 OR S33 | 200 |
| S33 | S27 AND S32 | 15 |
| S32 | S28 OR S29 OR S30 OR S31 | 145712 |
| S31 | (MH "Virus Diseases+") | 137287 |
| S30 | (MH "Viruses+") | 29364 |
| S29 | (MH "Toxoplasmosis+") | 875 |
| S28 | TORCH | 261 |
| S27 | (MH "Nervous System Abnormalities+/EM/EP/ET") | 781 |
| S26 | S11 AND S25 | 185 |
| S25 | S12 OR S13 OR S14 OR S15 OR S16 OR S17 OR S18 OR S19 OR S20 OR S21 OR S22 OR S23 OR S24 | 382515 |
| S24 | (prenatal or antenatal or fetus or fetal or foetus or foetal or gestation* or intrauter* or pregnan* or "expectant mother*" or stillbirth*) | 176976 |
| S23 | (cephalometr* or (head N3 (circumference* or size))) | 4217 |
| S22 | (microcephal* or microlissencephal* or anencephal* or ((congenital or brain or cerebral or "white matter" or nerv* or neur*) N3 (malformation* or abnormalit* or defect* or calcification or development* or growth)) or neurotroph*) | 24351 |
| S21 | (MH "Prenatal Diagnosis+") | 12212 |
| S20 | (MH "Prenatal Care") OR (MH "Prepregnancy Care") | 11885 |
| S19 | (MH "Expectant Mothers") | 2757 |
| S18 | (MH "Pregnancy+") | 138371 |
| S17 | (MH "Maternal Exposure") | 1376 |
| S16 | (MH "Cephalometry") | 2846 |
| S15 | (MH "Nerve Growth Factors+") | 856 |
| S14 | (MH "Embryonic Structures+") | 27678 |
| S13 | (MH "Growth and Embryonic Development+") | 117358 |
| S12 | (MH "Congenital, Hereditary, and Neonatal Diseases and Abnormalities+") | 121383 |
| S11 | S4 OR S10 | 4220 |
| S10 | S5 OR S6 OR S7 OR S8 OR S9 | 844 |
| S9 | arbovirus* | 485 |
| S8 | (MH "Arboviruses") | 44 |
| S7 | chikungunya | 382 |
| S6 | (MH "Chikungunya Fever") | 198 |
| S5 | (MH "Chikungunya Virus") | 144 |
| S4 | S1 or S2 or S3 | 3798 |
| S3 | (zika or flavivi* or "flavi vi*" or dengue or (encephalitis N3 (japan* or "st louis" or "tick borne")) or "west nile fever*" or "yellow fever*") | 3763 |
| S2 | (MH "Flavivirus Infections+") | 2983 |
| S1 | (MH "Flavivirus") | 45 |
