# Supplementary material for: Zika virus infection in pregnancy: a systematic review of disease course and complications
Source: Reprod Health. 2017 Feb 28;14:28. doi: 10.1186/s12978-017-0285-6 (PMC5330035; doi:10.1186/s12978-017-0285-6)
Supplement: Additional file 2: — List of excluded studies ZIKV & pregnancy RHER1. (DOCX 31 kb) [file 12978_2017_285_MOESM2_ESM.docx]

Additional file 2

| **No** | **Title** | **Reason for exclusion** |
| --- | --- | --- |
| **1** | Brasil, L.M., et al., Web platform using digital image processing and geographic information system tools: a Brazilian case study on dengue. Biomedical Engineering Online, 2015. 14: p. 69. | No primary data, paper discusses prevention strategy for dengue mosquitos which can be applied to ZIKV infection. |
| **2** | Grabowski, J.M., et al., Changes in the Proteome of Langat-Infected Ixodes scapularis ISE6 Cells: Metabolic Pathways Associated with Flavivirus Infection. PLoS Neglected Tropical Diseases [electronic resource], 2016. 10(2): p. e0004180. | Not related to review objective. |
| **3** | Gulland, A., WHO strengthens Zika travel advice to pregnant women. BMJ, 2016. 352: p. i1460. | No primary data, news comment/summary about WHO press conference on the ZIKV meeting |
| **4** | Goorhuis, A., et al., Zika virus and the risk of imported infection in returned travelers: Implications for clinical care. Travel Medicine & Infectious Disease, 2016. 14(1): p. 13-5. | No primary data, case reports on male and female seniors of >50 years with imported ZIKV infection from Suriname |
| **5** | Cover photo. Bulletin of the World Health Organization, 2016. 94(3): p. 162-162 1/9p. | No primary data, a commentary. |
| **6** | Brito, C., Zika virus: A new chapter in the history of medicine. Acta Medica Portuguesa, 2015. 28(6): p. 679-680. | No primary data, a narrative summary. |
| **7** | Petersen, E., et al., Rapid Spread of Zika Virus in The Americas - Implications for Public Health Preparedness for Mass Gatherings at the 2016 Brazil Olympic Games. International Journal of Infectious Diseases, 2016. 44: p. 11-5. | No primary data, opinion paper which discusses implications for ZIKV infection in mass gatherings like the Olympics. |
| **8** | Torjesen, I., Zika virus outbreaks prompt warnings to pregnant women. BMJ, 2016. 352: p. i500. | No primary data, news release |
| **9** | Chang, C., et al., The Zika outbreak of the 21st century. Journal of Autoimmunity, 2016. 68: p. 1-13. | No primary data, extensive narrative review on spread, mode of transmission, pathogenesis, prevention and treatment for ZIKV infection |
| **10** | Yakob, L. and T. Walker, Zika virus outbreak in the Americas: The need for novel mosquito control methods. The Lancet Global Health, 2016. 4(3): p. e148-e149. | No primary data, a lancet commentary |
| **11** | Heymann, D.L., et al., Zika virus and microcephaly: why is this situation a PHEIC? Lancet, 2016. 387(10020): p. 719-21. | No primary data, a commentary on ZIKV infection as PHEIC |
| **12** | Roa, M., Zika virus outbreak: reproductive health and rights in Latin America. 2016, Lancet: Philadelphia, Pennsylvania. p. 843-843 2/3p. | No primary data, an opinion article |
| **13** | Azevedo, A., Brazil's scientists scramble to solve the Zika puzzle. 2016, World Health Organization. p. 165-166 2p. | No primary data, news interview of Azevedo et al. who is currently following 300 women suspected to have ZIKV infection. |
| **14** | Heukelbach, J., et al., Zika virus outbreak in Brazil. Journal of Infection in Developing Countries, 2016. 10(2): p. 116-20. | No primary data, a review |
| **15** | Elachola, H., et al., A crucial time for public health preparedness: Zika virus and the 2016 Olympics, Umrah, and Hajj. Lancet, 2016. 387(10019): p. 630-2. | No primary data, a narrative review extending ZIKV effects to preparedness for international events |
| **16** | Higgs, S., Zika Virus: Emergence and Emergency. Vector-Borne and Zoonotic Diseases, 2016. 16(2): p. 75-76. | No primary data, an editorial comment on ZIKV infection |
| **17** | Lucey, D.R., Time for global action on Zika virus epidemic. BMJ: British Medical Journal, 2016. 352(8044): p. i781-i781 1p. | No primary data, an editorial |
| **18** | McCarthy, M., CDC updates Zika virus guidance to protect pregnant women. BMJ, 2016. 352: p. i786. | No primary data, a news release. |
| **19** | Mor, G., Placental Inflammatory Response to Zika Virus may Affect Fetal Brain Development. American Journal of Reproductive Immunology, 2016. 75(4): p. 421-2. | No primary data, a narrative review on postulated effects of ZIKV infection in neonates via placental transmission |
| **20** | Samarasekera, U. and M. Triunfol, Concern over Zika virus grips the world. The Lancet, 2016. 387(10018): p. 521-524. | No primary data, a review |
| **21** | Burke, R.M., et al., Zika virus infection during pregnancy: What, where, and why? British Journal of General Practice, 2016. 66(644): p. 122-123. | No primary data, an editorial reporting on history, spread and advice for prevention of ZKV infection |
| **22** | Byass, P. and A. Wilder-Smith, Utilising additional sources of information on microcephaly. 2016, Lancet: Philadelphia, Pennsylvania. p. 940-941 2p. | No primary data, a narrative summary on possible sources of information for microcephaly associated with ZIKV infection |
| **23** | Siqueira, W.L., et al., Zika virus infection spread through saliva - a truth or myth? Pesquisa Odontologica Brasileira = Brazilian Oral Research, 2016. 30(1): p. e46. | No primary data, opinion article |
| **24** | MacFadden, D.R. and I.I. Bogoch, Zika virus infection. CMAJ: Canadian Medical Association Journal, 2016. 188(5): p. 367-367 1p. | No primary data, a fact sheet |
| **25** | Ayres, C.F.J., Identification of Zika virus vectors and implications for control. The Lancet Infectious Diseases, 2016. 16(3): p. 278-279. | No primary data, a narrative summary on the possibility of vectors other than A. aegypti for ZIKV spread |
| **26** | McCarthy, M., Zika virus outbreak prompts US to issue travel alert to pregnant women. BMJ, 2016. 352: p. i306. | No primary data, a news release |
| **27** | Vouga, M., et al., CDC guidelines for pregnant women during the Zika virus outbreak. 2016, Lancet: Philadelphia, Pennsylvania. p. 843-844 2p. | No primary data, a review providing suggestions on when to perform amniocentesis |
| **28** | Vogel, G., A race to explain Brazil's spike in birth defects : Evidence points toward the fast-spreading Zika virus as the cause of microcephaly. Science, 2016. 351(6269): p. 110-111. | No primary data, a news release |
| **29** | Dyer, O., SIXTY SECONDS ON . . . ZIKA VIRUS. BMJ: British Medical Journal, 2016. 352(8042): p. 129-129 1/2p. | No primary data, a news release |
| **30** | Dyer, O., Jamaica advises women to avoid pregnancy as Zika virus approaches. BMJ, 2016. 352: p. i383. | No primary data, a news report |
| **31** | Stratton, S.J., Zika Virus Association with Microcephaly: The Power for Population Statistics to Identify Public Health Emergencies. Prehospital & Disaster Medicine, 2016. 31(2): p. 119-20. | No primary data, a viewpoint article on the importance of epidemiology |
| **32** | Mayor, S., Zika infection in pregnancy is linked to range of fetal abnormalities, data indicate. BMJ, 2016. 352: p. i1362. | This study provides primary data on the same cohort of women in one of the studies included in the review (Brasil P. et al), to avoid repetition we excluded it |
| **33** | Millichap, J.G., Zika Virus Infection and Microcephaly. Pediatric Neurology Briefs, 2016. 30(1): p. 8. | This study provides primary data on the same cohort of women in one of the studies included in the review (Schuler Faccini et al.) To prevent double reporting we excluded it. |
| **34** | Kelvin, A.A., et al., ZIKATracker: A mobile App for reporting cases of ZIKV worldwide. Journal of Infection in Developing Countries, 2016. 10(2): p. 113-5. | No primary data, a report on the development of a mobile application to report ZIKV infected cases. |
| **35** | Ginier, M., et al., Zika without symptoms in returning travellers: What are the implications? Travel Medicine and Infectious Disease, 2016. 14(1): p. 16-20. | No primary data, a case report in a non-pregnant woman |
| **36** | Carneiro, L.A.M. and L.H. Travassos, Autophagy and viral diseases transmitted by Aedes aegypti and Aedes albopictus. Microbes and Infection, 2016. 18(3): p. 169-171. | No primary data, a review |
| **37** | Iacobucci, G., Zika highlights need for ethical framework for developing vaccines for pregnant women. BMJ, 2016. 352: p. i1155. | No primary data, a news release/ comment on an article about vaccination for pregnant women and Zika (Omer 2016) |
| **38** | Malone, R.W., et al., Zika Virus: Medical Countermeasure Development Challenges. PLoS Neglected Tropical Diseases [electronic resource], 2016. 10(3): p. e0004530. | No primary data, an epidemiological, survey, narrative review and analysis on spread, countries affected, projections. A review which analyzes information concerning the ZIKV outbreak in South America, Central America, and the Caribbean and the status of relevant medical countermeasures (MCM) available for treating or preventing Zika virus infection and disease. |
| **39** | Attar, N., ZIKA virus circulates in new regions. Nature Reviews Microbiology, 2016. 14(2): p. 62. | No primary data, a narrative summary on Zika spread |
| **40** | Rodriguez-Morales, A.J., Zika and microcephaly in Latin America: An emerging threat for pregnant travelers? Travel Medicine & Infectious Disease, 2016. 14(1): p. 5-6 2p. | No primary data, an editorial |
| **41** | Cofre, F., [Zika virus intrauterine infection causes fetal brain abnormality and microcephaly: tip of the iceberg?]. Revista Chilena de Infectologia, 2016. 33(1): p. 96. | No primary data. This article reports 2 cases, however, it’s a translated version of the Oliveira Melo et al. article, therefore not an actual report. |
| **42** | Barreto, M.L., et al., Zika virus and microcephaly in Brazil: a scientific agenda. Lancet, 2016. 387(10022): p. 919-21. | No primary data, the study refers to a review by Soares et al and suggests an action plan for the government relative to ZIKV infection |
| **43** | Victora, C.G., et al., Microcephaly in Brazil: How to interpret reported numbers? The Lancet, 2016. 387(10019): p. 621-624. | No primary data |
| **44** | Wong, S.S.Y., R.W.S. Poon, and S.C.Y. Wong, Zika virus infection-the next wave after dengue? Journal of the Formosan Medical Association, 2016((Wong S.S.Y., samsonsy@hku.hk) Department of Microbiology, Research Centre for Infection and Immunology, Faculty of Medicine, The University of Hong Kong, Hong Kong). | No primary data, the paper provides detailed information on ZIKV infection |
| **45** | Von Eije, K.J., et al., Imported Zika virus infection in the Netherlands. Nederlands Tijdschrift voor Geneeskunde, 2016. 160(8). | No primary data, a news commentary |
| **46** | Villa, R., Zika, or the burden of uncertainty. Clinica Terapeutica, 2016. 167(1): p. 7-9. | No primary data, an editorial |
| **47** | Turnbul, A., Midwives urged to advise pregnant women on Zika. Independent Nurse, 2016: p. 2-2 1p. | No primary data |
| **48** | Teixeira, M.G., et al., The Epidemic of Zika Virus--Related Microcephaly in Brazil: Detection, Control, Etiology, and Future Scenarios. American Journal of Public Health, 2016. 106(4): p. 601-605 5p. | No primary data, a description of the epidemiology of ZIKV |
| **49** | Talan, J., Epidemiologists Are Tracking Possible Links Between Zika Virus, Microcephaly, and Guillain—Barre Syndrome. Neurology Today, 2016. 16(4): p. 1-23 7p. | No primary data, a news item |
| **50** | Staples, J.E., et al., Interim Guidelines for the Evaluation and Testing of Infants with Possible Congenital Zika Virus Infection - United States, 2016. MMWR - Morbidity & Mortality Weekly Report, 2016. 65(3): p. 63-7. | No primary data, an interim guideline for health care providers in the US developed by CDC |
| **51** | Sikka, V., et al., The emergence of zika virus as a global health security threat: A review and a consensus statement of the INDUSEM Joint working Group (JWG). Journal of Global Infectious Diseases, 2016. 8(1): p. 3-15. | No primary data, a detailed narrative review |
| **52** | Saxena, S.K., et al., Zika virus outbreak: an overview of the experimental therapeutics and treatment. VirusDisease, 2016((Saxena S.K., shailen@ccmb.res.in; Elahi A.; Gadugu S.; Prasad A.K.) CSIR-Centre for Cellular and Molecular Biology (CCMB), Hyderabad, India): p. 1-5. | No primary data, an overview on experimental treatments associated with the ZIKV outbreak |
| **53** | Rubin, E.J., M.F. Greene, and L.R. Baden, Zika Virus and Microcephaly. The New England journal of medicine, 2016. 374(10): p. 984-985. | No primary data, a narrative review providing narrative information, the review also suggests no link between microcephaly and ZIKV infection |
| **54** | Rodriguez-Morales, A.J., A.C. Bandeira, and C. Franco-Paredes, The expanding spectrum of modes of transmission of Zika virus: A global concern. Annals of Clinical Microbiology and Antimicrobials, 2016. 15(1). | No primary data, an editorial |
| **55** | Petersen, E.E., et al., Interim Guidelines for Pregnant Women During a Zika Virus Outbreak - United States, 2016. MMWR - Morbidity & Mortality Weekly Report, 2016. 65(2): p. 30-3. | No primary data, CDC recommendations for travelling pregnant women |
| **56** | Paixão, E.S. and L.C. Rodrigues, What we need to know about Zika virus. British Journal of Hospital Medicine (17508460), 2016. 77(3): p. 124-125 2p. | No primary data, an editorial |
| **57** | Paixao, E.S., et al., History, Epidemiology, and Clinical Manifestations of Zika: A Systematic Review. American Journal of Public Health, 2016. 106(4): p. 606-12. | No primary data, a systematic review on general ZIKV infection with no isolated data for pregnant women. |
| **58** | Oster, A.M., et al., Interim Guidelines for Prevention of Sexual Transmission of Zika Virus - United States, 2016. MMWR - Morbidity & Mortality Weekly Report, 2016. 65(5): p. 120-1. | No primary data, CDC guidelines for prevention of sexual transmission |
| **59** | Omer, S.B. and R.H. Beigi, Pregnancy in the Time of Zika: Addressing Barriers for Developing Vaccines and Other Measures for Pregnant Women. JAMA, 2016. 315(12): p. 1227-8. | No primary data, study reports on the barriers for vaccine development for pregnant women |
| **60** | Olsen, B. and A. Lundkvist, [In Process Citation]. Lakartidningen, 2016. 113. | No primary data, there were no cases of ZIKV infected pregnancies |
| **61** | Oduyebo, T., et al., Update: Interim Guidelines for Health Care Providers Caring for Pregnant Women and Women of Reproductive Age with Possible Zika Virus Exposure -- United States, 2016. MMWR: Morbidity & Mortality Weekly Report, 2016. 65(5): p. 122-127 6p. | No primary data, CDC guideline updates for clinicians |
| **62** | Mohamad Idris, F., Zika – A pandemic in progress? Malaysian Journal of Medical Sciences, 2016. 23(2): p. 70-72. | No primary data, the study narratively discusses Aedes agent of ZIKV infection and the possibility of adaptation to other Aedes vectors |
| **63** | Miranda-Filho, D.d.B., et al., Initial Description of the Presumed Congenital Zika Syndrome. American Journal of Public Health, 2016. 106(4): p. 598-600. | No primary data, an overview which describes the concept of presumed congenital Zika syndrome |
| **64** | Mende, A., Zika virus in South America: Travel guidelines for pregnant women. Pharmazeutische Zeitung, 2015. 160(49). | No primary data, a guideline for travelers relative to ZIKV infection |
| **65** | McDonnell, P.J., Playing 'hide and Zika'. Ophthalmology Times, 2016. 41(4): p. 6-6 2/3p. | No primary data, an editorial |
| **66** | Maurice, J., WHO reveals its shopping list for weapons against Zika. The Lancet, 2016. 387(10020): p. 733. | No primary data, the article describes WHO action plan to stem ZIKV spread, tests, drugs and vaccines in the pipeline |
| **67** | Martines, R.B., et al., Notes from the Field: Evidence of Zika Virus Infection in Brain and Placental Tissues from Two Congenitally Infected Newborns and Two Fetal Losses - Brazil, 2015. MMWR - Morbidity & Mortality Weekly Report, 2016. 65(6): p. 159-60. | To avoid double reporting, we excluded this study. This CDC report provides data on the same cohort mentioned in CDC MMWR report. It discusses evidence of a link between Zika virus infection and microcephaly and fetal demise through detection of viral RNA and antigens in brain tissues from infants with microcephaly and placental tissues from early miscarriages. |
| **68** | Marrs, C., et al., Zika Virus and Pregnancy: A Review of the Literature and Clinical Considerations. American Journal of Perinatology, 2016((Marrs C., carolinemarrs@gmail.com; Olson G.; Saade G.; Hankins G.; Wen T.) Division of Maternal-Fetal Medicine, Department of Obstetrics and Gynecology, The University of Texas Medical Branch, Galveston, Texas). | No primary data, a narrative review which discusses Zika virus in pregnancy |
| **69** | Lupton, K., Zika virus disease: a public health emergency of international concern. British Journal of Nursing, 2016. 25(4): p. 198-202. | No primary data, the study provides a narrative summary of signs, symptoms, prevention, treatment and travel advice all related to ZIKV infection. |
| **70** | Lucey, D.R. and L.O. Gostin, The Emerging Zika Pandemic: Enhancing Preparedness. JAMA, 2016. 315(9): p. 865-6. | No primary data, a viewpoint article which provides tips on preparation strategies relative to ZIKV infection |
| **71** | Lucey, D.R., Time for global action on Zika virus epidemic: Our response to infectious disease epidemics must be faster and smarter. BMJ (Online), 2016. 352((Lucey D.R., DRL23@Georgetown.edu) Department of Medicine, Infectious Diseases, Georgetown University Medical Center, Washington, United States). | No primary data, an editorial summary |
| **72** | Lockwood, C.J., Zika virus and microcephaly. Contemporary OB/GYN, 2016. 61(2): p. 6-9 3p. | No primary data, an opinion article discussing the magnitude of ZIKV infection threat and the MCP-ZIKV link. |
| **73** | Li, J.D. and D.X. Li, [Epidemiological characteristics of Zika virus disease]. Chung-Hua Liu Hsing Ping Hsueh Tsa Chih Chinese Journal of Epidemiology, 2016. 37(3): p. 329-34. | No primary data, an epidemiologic systematic review in Chinese |
| **74** | Laoprasopwattana, K., et al., Chikungunya and dengue virus infections during pregnancy: seroprevalence, seroincludeidence and maternal-fetal transmission, southern Thailand, 2009-2010. Epidemiology & Infection, 2016. 144(2): p. 381-8. | No primary data, a report on chikungunya and dengue virus infection, no co-infection with Zika was reported |
| **75** | Krader, C.G., Zika virus and Chikungunya in the U.S.--Syphilis infections includerease in newborns. Dermatology Times, 2016. 37(3): p. 10-18 2p. | No primary data, a news article on ZIKV infection |
| **76** | Korhonen, E.M., et al., Zika virus infection in a traveller returning from the Maldives, June 2015. Euro Surveillance: Bulletin Europeen sur les Maladies Transmissibles = European Communicable Disease Bulletin, 2016. 21(2): p. 14. | No primary data, a case report on the history of ZIKV infection in a male patient. Initially negative, 2 weeks later he had a low dengue viral seropositivity attributed to cross reactivity between flaviviruses |
| **77** | Hills, S.L., et al., Transmission of Zika Virus Through Sexual Contact with Travelers to Areas of Ongoing Transmission - Continental United States, 2016. MMWR - Morbidity & Mortality Weekly Report, 2016. 65(8): p. 215-6. | No primary data, case reports on only males and no pregnant cases |
| **78** | Hibbeler, B., Zika virus infections: Risk for pregnant women. Deutsches Arzteblatt International, 2016. 113(4): p. A137. | No primary data, a news article on ZIKV infection |
| **79** | Hennigan, T., Brazil struggles to cope with Zika epidemic. BMJ (Online), 2016. 352((Hennigan T., hennigantom@gmail.com) São Paulo, Brazil). | No primary data, a news report |
| **80** | Hasson, M., K.L. Kahl, and T. Nhu, Vision-threatening abnormalities possible in infants with presumed Zika virus-associated microcephaly. Ocular Surgery News, 2016. 34(5): p. 25-25 1/3p. | Duplicate of de Paula Freitas et al, one of the included studies. We excluded this study to prevent double reporting |
| **81** | Gulland, A., Genetically modified mosquitos may be used in fight against Zika. BMJ: British Medical Journal, 2016. 352(8046): p. 298-299 2p. | No primary data, a news report |
| **82** | Godlee, F., Zika, and rapid diagnostic tests for malaria. BMJ (Online), 2016. 352((Godlee F., fgodlee@bmj.com) BMJ, United States). | No primary data, a news report |
| **83** | Gatherer, D. and A. Kohl, Zika virus: a previously slow pandemic spreads rapidly through the Americas. Journal of General Virology, 2016. 97(2): p. 269-73. | No primary data, a narrative review which discusses phylogenetic links with ZIKV infection |
| **84** | Galindo-Fraga, A., et al., Zika Virus: A New Epidemic on Our Doorstep. Revista de Investigacion Clinica, 2015. 67(6): p. 329-32. | No primary data, a narrative review which provides a comparison of the main clinical manifestations of dengue, chikungunya, and Zika |
| **85** | Fleming-Dutra, K.E., et al., Update: Interim Guidelines for Health Care Providers Caring for Infants and Children with Possible Zika Virus Infection - United States, February 2016. MMWR - Morbidity & Mortality Weekly Report, 2016. 65(7): p. 182-7. | No primary data, a CDC guideline |
| **86** | Fauci, A.S. and D.M. Morens, Zika Virus in the Americas--Yet Another Arbovirus Threat. New England Journal of Medicine, 2016. 374(7): p. 601-604 4p. | No primary data, a narrative review on the history of ZIKV infections |
| **87** | Evans, G., Zika Questions Abound, but Standard Precautions Will Stop it. Hospital Infection Control & Prevention, 2016. 43(3): p. 25-29 5p. | No primary data, a viewpoint article |
| **88** | Dyer, O., Zika virus spreads across Americas as concerns mount over birth defects. BMJ (Clinical research ed.), 2015. 351((Dyer O.) Montreal): p. h6983. | No primary data, a news report |
| **89** | Dhimal, M., et al., Zika Virus: Yet Another Emerging Threat to Nepal. Journal of Nepal Health Research Council, 2015. 13(31): p. 248-51. | No primary data, a narrative review |
| **90** | Check Hayden, E., Spectre of Ebola haunts Zika response. Nature, 2016. 531(7592): p. 19. | No primary data, a commentary |
| **91** | Cetron, M., Revision to CDC's Zika Travel Notices: Minimal Likelihood for Mosquito-Borne Zika Virus Transmission at Elevations Above 2,000 Meters. MMWR - Morbidity & Mortality Weekly Report, 2016. 65(10): p. 267-8. | No primary data, a narrative review |
| **92** | Butler, D., Zika virus: Brazil's surge in small-headed babies questioned by report. Nature, 2016. 530(7588): p. 13-14. | No primary data, a nature review article |
| **93** | Brown, C., Zika virus outbreaks in Asia and South America. CMAJ: Canadian Medical Association Journal, 2016. 188(2): p. E34-E34 1p. | No primary data, a news report |
| **94** | Blonz, E.R. and C.J. Lockwood, Zika and glucose levels...Lockwood CJ. Zika virus and microcephaly. Contemporary OB/GYN 2016 Feb 61(2):6-9. 2016, Advanstar Communications Include.: North Olmsted, Ohio. p. 12-14 2p. | No primary data, the study proposes a link between GLU1 transport deficiency and microcephaly all mitigated by ZIKV infection. |
| **95** | Bell, B.P., C.A. Boyle, and L.R. Petersen, Preventing Zika Virus Infections in Pregnant Women: An Urgent Public Health Priority. American Journal of Public Health, 2016. 106(4): p. 589-90. | No primary data, an editorial which discusses progression and history and ZIKV infection |
| **96** | Basarab, M., et al., Zika virus. BMJ (Online), 2016. 352((Basarab M.; Bowman C.; Cropley I., iancropley@nhs.net) Department of Infectious Diseases, Royal Free London NHS Foundation Trust, London, United Kingdom). | No primary data, a clinical review |
| **97** | Ahmad, S.S.Y., T.N. Amin, and A. Ustianowski, Zika virus: Management of infection and risk. BMJ (Online), 2016. 352((Ahmad S.S.Y.; Ustianowski A., Andrew.Ustianowski@pat.nhs.uk) Northwest Regional Infectious Diseases Unit, North Manchester General Hospital, Pennine Acute Hospitals Trust, Manchester, United Kingdom). | No primary data, study suggests management for potential clinical scenarios of ZIKV infection |
| **98** | Pyriproxyfen, Zika and Microcephaly. Positive Health, 2016(229): p. 11-11 1p. | No primary data, a short report linking pyripoxifen from fertilizer usage to microcephaly incidence rather than maternal ZIKV infection |
| **99** | Zika Virus, Microcephaly, Guillain-Barré, Genetically Modified Mosquitos and Potential Vaccine Damage. Positive Health, 2016(229): p. 12-12 1p. | No primary data, a study suggesting prevention strategies |
| **100** | Zika: A clinical guide. 2016, BMJ Publishing Group. p. 366-366 1p. | No primary data, the study provides a conceptual diagram relative to ZIKV infection |
| **101** | LATEST FREE ZIKA VIRUS RESOURCES FROM BMJ. BMJ: British Medical Journal, 2016(8045): p. 262-262 1/3p. | No primary data, a collation of reader responses on ZIKV |
| **102** | Zika Virus: what you need to know. Midwifery Matters, 2016(148): p. 3-3 1/2p. | No primary data, an abridged educational report on ZIKV infection |
| **103** | Advice on Zika virus. Primary Health Care, 2016. 26(2): p. 7-7 1/5p. | No primary data |
| **104** | Science committee calls on government to build Zika virus evidence base. Emergency Nurse, 2016. 23(10): p. 6-6 1/4p. | No primary data, a news article on ZIKV infection |
| **105** | Zika 101: Prime threat for the pregnant. Hospital Employee Health, 2016. 35(3): p. 32-34 3p. | No primary data, a CDC educational report for the public related to ZIKV infection |
| **106** | Public health authorities race to contain fast-moving Zika outbreak. ED Management, 2016. 28(4): p. 37-41 5p. | No primary data |
| **107** | NEWS ROUND-UP. Community Practitioner, 2016. 89(3): p. 6-7 2p. | No primary data, a news release discussing WHO prediction of ZIKV affecting 4 million |
| **108** | Zika virus: A new global threat for 2016. The Lancet, 2016. 387(10014): p. 96. | No primary data, the study discusses the history of ZIKV infection |
| **109** | The next steps on Zika. Nature, 2016. 530(7588): p. 5. | No primary data, the study provides suggestions on actions to take relative to ZIKV infection |
| **110** | Resources and latest news about zika virus disease available from ECDC. Eurosurveillance, 2016. 21(5). | No primary data, an editorial summarizing ZIKV related information |
| **111** | European Commission Horizon 2020 programme call for vaccine development research into malaria and neglected infectious diseases, including Zika virus. Eurosurveillance, 2016. 21(6). | No primary data, an editorial calling for proposals on research and vaccinations related to ZIKV infection |
| **112** | Zika virus in the dock. The Lancet Infectious Diseases, 2016. 16(3): p. 265. | No primary data, the study provides narrative information relative to general ZIKV infection |
| **113** | Zika virus infection: global update on epidemiology and potentially associated clinical manifestations. Weekly Epidemiological Record, 2016. 91(7): p. 73-81. | No primary data, a narrative review and update on general epidemiology and potential clinical manifestations for ZIKV infection |
| **114** | Healthcare staff encouraged to warn patients of the risks of the Zika virus. Nursing Standard, 2016. 30(24): p. 9. | No primary data, a news report discussing links to Zika virus infection, guidance for primary care and clinicians |
| **115** | Zika virus. Nursing Standard, 2016. 30(24): p. 17. | No primary data, clinical updates on ZIKV infection |
| **116** | Musso, D. and D. Baud, Zika virus: Time to move from case reports to case control. The Lancet Infectious Diseases, 2016((Musso D., dmusso@ilm.pf) Unit of Emerging Infectious Diseases, Institut Louis Malardé, PO Box 30, 98713 Papeete, Tahiti, French Polynesia) | No primary data, the study advocates a move from case studies to case-control all in the context of ZIKV infection |
| **117** | Epidemiological report of week 8 monitoring cases of microcephaly in Brazil | No primary data |
| **118** | Protocol for surveillance and response to the occurrence of microcephaly related to zika virus infection | No primary data |
| **119** | Technical report: possible change in the epidemiological pattern of microcephaly in Pernambuco | No primary data |
| **120** | Clinical and epidemiological protocol for investigating cases of microcephaly in the state of Pernambuco | No primary data |
| **121** | Zika virus: information to the public | No primary data |
| **122** | Brazilian Ministry of Health. 12 January 2016: Weekly epidemiological update on suspected microcephaly cases (Novos casos suspeitos de microcefalia são divulgados pelo Ministério da Saúde) (12 January 2016) Available at: http:// portalsaude.saude.gov.br/index.php/cidadao/princludeipal/agencia-saude/ 21677-novos-casos-suspeitos-de-microcefalia-sao-divulgados-pelo- ministerio-da-saude –accessed January 26th, 2016. | No primary data |
| **123** | Hazin, A. N., Poretti, A., Cruz, D. D. C. S., Tenorio, M., van der Linden, A., Pena, L. J., ... & Martelli, C. M. T. (2016). Computed tomographic findings in microcephaly associated with Zika virus. *New England Journal of Medicine*. | No primary data |
| **124** | Petersen, L. R., Jamieson, D. J., Powers, A. M., & Honein, M. A. (2016). Zika Virus. *New England Journal of Medicine*. | No primary data |
